# Royal Academy of Medicine in Ireland

**Published:** 1888-03

**Authors:** 


ROYAL ACADEMY OF MEDICINE IN IRELAND.

*The Surgery of the Thyroid Gland.*—Mr. FOY read a paper on the surgery of the thyroid gland. After tracing the history of operations for the ablation of the gland and extirpation of tumor, he compared the modern operation of Dr. P. H. Watson with that recommended and practiced by Desault at the Hôtel Dieu in 1791. He condemned the many minor operations of setons, caustics, injections of irritants, and tapping, and gave the history of a successful removal of a cysto-adenoma from the right lobe of a young married woman's thyroid.

The PRESIDENT said that Dr. P. H. Watson had advocated the operation for the complete removal of the gland, while at the same time indicating that the surgeon must be prepared to see the patient die on the table—a fatality which occurred at least once in that distinguished surgeon's practice; and he dwelt strongly on the necessity of leaving the capsule untorn, especially the capsule surrounding the vessels.

Mr. STORY inquired what the indications were for operating on tumor on the thyroid gland.

Mr. KENDAL FRANKS did not think the size of a tumor in the neck a guide to operative procedure. Small tumors indicating a tendency to press backwards and sometimes down under the top of the sternum caused great dyspnœa and endangered life.

Mr. W. THORNLEY STOKER had operated in several cases of goitre, both by removal and by division of the isthmus, and in his experience the operator must be prepared for terrible hæmorrhage. He condemned, with Mr. Franks, the practice of passing a seton through the gland. The *rationale* of the operation of the division of the isthmus was that it limited the blood supply of the gland. He found it gave relief for the time and set the trachea free. As regarded the opening of the capsule he had no decided opinion, being unable in his operations to find out where or what it was; for, when he cut down on the diseased structure, he came on the gland covered by enormous veins, some as big as his thumb, and whether these were inside or outside the capsule he had not been able to determine.

The PRESIDENT said that thirteen years ago he had a case of acute goitre, and, at the suggestion of Dr. Purser, he had given five or ten grains of quinine three times a day, and in a week or ten days the growth stopped. Happening to be with Sir William MacCormac in St. Thomas's Hospital he saw a patient with a tumor on the front of the neck that grew rapidly, and he mentioned the quinine cure. Sir William MacCormac tried it, and in fourteen days the growth ceased and the patient recovered.

Mr. Foy, in reply, considered that the operation was justified when the tumor was growing quickly, when dyspnœa or dysphagia was marked, or when any evidence of malignancy was present, and also when the most improved internal and external medication had not given good results. In the swampy districts of the Carolinas a malarial type of goitre was prevalent which was amenable to treatment by quinine; but the occurrence of such cases in this country must be very rare. Setons, tapping, and caustics were not free from danger, and in many cases did not give favorable results. As for Sir Morell Mackenzie's method of injecting perchloride of iron, he mentioned it only as a treatment most unsuitable and to be avoided.

